# Supplementary material for: Patterns of Intron Gain and Loss in Fungi
Source: PLoS Biol. 2004 Nov 30;2(12):e422. doi: 10.1371/journal.pbio.0020422 (PMC532390; doi:10.1371/journal.pbio.0020422)
Supplement: Table S1 — Also available at http://genes.mit.edu/NielsenEtAl/. (4.3 MB ZIP). [file pbio.0020422.st001.zip › NielsenEtAl/html/709.html]

AN3869.1.NCU03633.1.MG06946.1.FG05912.1


```
 CLUSTAL W (1.82) Multiple Sequence Alignments - Introns Inserted


Sequence 1: NCU03633.1	528 aa
Sequence 2: FG05912.1	508 aa
Sequence 3: MG06946.1	494 aa
Sequence 4: AN3869.1	735 aa
Alignment Length: 739 aa
Number Identitical Residues: 252 aa
Alignment Score (without introns) 12579


MG06946.1 	----------------------~-------------------------------------
NCU03633.1	----------------------~-------------------------------------
FG05912.1 	----------------------~-------------------------------------
AN3869.1  	MASYLLLLLHRDFVLSCLVPYF1GYKLTYRTKFCDPRTVDLQPGRRPLGVKEIKELDDYK
          	 ::        .   :.  .   . . :  :. ... : . ...  . . .. .. .. .

MG06946.1 	---------------~-------------------~------------------~-----
NCU03633.1	---------------~-------------------~------------------~-----
FG05912.1 	---------------~-------------------~------------------~-----
AN3869.1  	SIAPWRESVSFVQLC2RERAMYTRIDLELLQPQYN0TSCPVTAVSFRCRNPGEM0SCHSG
          	: :.  .: :  . .  . :  :  . .  ... . ::.. :: :  . ....  :. :.

MG06946.1 	---------------------------------~--------------------------
NCU03633.1	---------------------------------~--------------------------
FG05912.1 	---------------------------------~--------------------------
AN3869.1  	DDAVERATGTMDNRIRARLTKRRYLDHDWWSLS1SSTRSLVRLGVILQLCAVLLPSFRLT
          	..: . ::.: ..   :  :.    . .  : : ::: :    .   . .:   .:   :

MG06946.1 	-----------------------------------MPP-TTVTTNGIHKEVG--ELHING
NCU03633.1	-----------------------------------MAEQEHNGVNGFHSESEQRNQPVNG
FG05912.1 	-----------------------------------MPPSNPAMVNGLNG------SHANG
AN3869.1  	ASQSWSLPDHINTPVFCSHSLLPAARTSYDLSLKLIEPYTLSTASDMTSLLPVRPRSVRP
          	::.: : ..  .:.  .: :  .:: :: . : . :       ...:           . 

MG06946.1 	------NGNG-------IVKNQ------------------RARMARKQSSPMMPAFMVSA
NCU03633.1	DASEAVNGNPSNGLRVTIEESASSAVNGGSPTNSMLT-PIRQRMERKKSSPMMPTFMVSA
FG05912.1 	------NGNGHNHISDSGSETSGESSNGSGRR--------RMKLNRKMSSPMAPPFMVSA
AN3869.1  	TAPVTISDDHESSDASTIPSPSTTTSSRSFSNPSTTPDQKRIKMQRKQSSPLAPAFMVSA
          	 :. : ..:  .    :  .    : . .    :  ..  * :: ** ***: *.*****

MG06946.1 	PGKVIVFGEHSVVYGK0KAVAAAINLRSYLLVTALSKSKRTITLRFPDIDLIHTWNIDDL
NCU03633.1	PGKVIVFGEHAVVHGK0AAIAAAISLRSYLLVNTLSKSKRTVTLKFPDIDFNHSWNIDEL
FG05912.1 	PGKVIVFGEHSVVHGK0AAIAAAISLRSYLHVTTLSKSKRTVSLRFADIGLVHTWNIEDL
AN3869.1  	PGKVIVYGEHAVVHGK~AAMAAAISLRSYLLVTTLSKSQRTITLNFRDTNLNHTWDIDQL
          	******:***:**:**  *:****.***** *.:****:**::*.* * .: *:*:*::*

MG06946.1 	PWSTFSQPSKKKYYYDLVTSLDPDLMDAIQPHLEPVSSDAPDAQRKVHMSAAAAFLYLFM
NCU03633.1	PWKIFQQPGKKKYYYSLVTEIDQELVDAVQPFLADVSIDKPADIRKVHQNSAGSFLYMFL
FG05912.1 	PWEAFQQPSKKKSYYSLVTELDPDLVAAIQPHIEVVSPNHPEEIRRVHHSSVSAFLYLFL
AN3869.1  	PWDLFHHPSKKKYYYDLVTTLDQELYDAIQPHVEDISPDAPEEMRKIHKRSASAFLYLFL
          	**. * :*.*** **.*** :* :*  *:**.:  :* : *   *::*  :..:***:*:

MG06946.1 	SLGSHAFPGGIYTLRSTIPIGAGLGSSASISACLSAALLLQIRTLSGPHPDQPPDEARVQ
NCU03633.1	SLGSQSFPGCQYTLRSTIPIGAGLGSSATIAVCLSAALLLQLRTLSGPHPDQPPEEARLQ
FG05912.1 	SLGSPSFPPCLYTLRSTIPIGAGLGSSASVSVCLASALLLQLRTLSGPHPDQPADEARLQ
AN3869.1  	SLGSPQSAGAIYTLRSTIPIGAGLGSSASVCVCMSAALLVQIRTLAGPHPDQPPDEAEVQ
          	****   .   *****************::..*:::***:*:***:*******.:**.:*

MG06946.1 	VERINRWAFVAEMCIHGNPSGVDNTVSTQGKAVVFQRLDYARPPVVTPMWDFPELPLLVV
NCU03633.1	IERINRWAFVYEMFIHGNPSGVDNTVSTQGKAVVFQRTDYNQPPSVRPLWDFPKLPLLLV
FG05912.1 	VERINRWAFVSEMCIHGNPSGVDNTVATQGKAVVFQRTDYSKPPNVRPLWDFPELPLLLV
AN3869.1  	IERINRWAFVGEMCIHGNPSGVDNTVSAGGKAVIFRRGDYSKPPSVTSLPTFPELPLLLV
          	:********* ** ************:: ****:*:* ** :** * .:  **:****:*

MG06946.1 	DTKQAKSTKYEVEKVAKLRETHPKIVNSILDSMDKLTQAATDVLTDEDFDNEDVESLQKV
NCU03633.1	DTRTAKSTAHEVAKVATLKKKHPQLVGTILTAIDQVTQSSAQLIEEQGFNTEDEESLSKV
FG05912.1 	DTRQAKSTAHEVAKVAKLKQTHPKLVNSILDAMDKVTDAASELIEETSFDNGSVEDLSKV
AN3869.1  	DTRQGRSTAVEVAKVGALRKEWPVVTESILDAIDNVTASAEKVIREPNPEGLSEKTLQQL
          	**: .:**  ** **. *::  * :. :** ::*::* :: .:: : . :  . : *.::

MG06946.1 	GELMGMNHGLLVSLGVSHPRLERVRELVDHEGIGWTKLTGAGGGGCSITLMRPDVPREKL
NCU03633.1	GEMMTINHGLLVSLGVSHPRLERVRELVDHEGIGWTKLTGAGGGGCSITLLRPGVPREKL
FG05912.1 	GELMTINHGLLVSLGVSHPRLERVRELVDHEGIGWTKLTGAGGGGCSITLLRPDVPAEKL
AN3869.1  	GTLIRINHGFLVSLGVSHPRLERIRELVDYADIGWTKLTGAGGGGCAITLLRPDLNDDSL
          	* :: :***:*************:*****: .**************:***:**.:  :.*

MG06946.1 	ERLKERLDHEGYLKFRTTLGGGGVGVLWPAVLRNGMDEDDEGGMEIDVEKFLNADGNEGV
NCU03633.1	DKLEQRLDEEGYSKFETTLGSDGVGVLWPAVLKNGMDEDEEGGMEIDLEKFLSADSNEAL
FG05912.1 	QKLEERLETENYAKFETTLGGDGIGVLWPAVLKNGTEEDEEGGMEIDLEKFLEAEGTEGV
AN3869.1  	RQLKSQLQDENFGIYETTLGGDGVGVLWPAVLRNGTDE--EGGEEIDQQKFENAVGTEGI
          	 :*:.:*: *.:  :.****..*:********:** :*  *** *** :** .* ..*.:

MG06946.1 	ERLVGVHGTSGEREGWKFWRVGNA-
NCU03633.1	EKLVGVHGDRGEREGWKFWRVENRD
FG05912.1 	EKLVGVHGDTGEREGWKFWRVESQ-
AN3869.1  	ERLVGVG-VQEKREGWKFWKRAVA-
          	*:****     :*******:
```
